# A wet chemical extraction protocol for measuring biogenic silica in sediments of marginal seas and open ocean

**DOI:** 10.1016/j.mex.2025.103723

**Published:** 2025-11-13

**Authors:** Dongdong Zhu, Su Mei Liu, Aude Leynaert, Paul Tréguer, Morgane Gallinari, Heting Zhou, Jill N. Sutton

**Affiliations:** a Frontiers Science Center for Deep Ocean Multi-spheres and Earth System, Key Laboratory of Marine Chemistry Theory and Technology, Ministry of Education, Ocean University of China, Qingdao 266100, China; b University of Brest, Centre national de la recherche scientifique, Institut de recherche pour le développement, Ifremer, Institut Universitaire Européen de la Mer, Plouzané 29280, France

**Keywords:** Analytical method, Opal, Marine sediments, Lithogenic silica, Silicon cycle

## Abstract

This study describes a wet chemical extraction protocol for measuring the biogenic silica (bSi) in sediments from diverse marine environments. The protocol lists the reagents, materials, equipment, and sample preparation procedures, and provides a detailed explanation of the methods for examining the alkaline-leachable silicon (Si), and calculating bSi content. Although, the protocol was primarily developed for measuring bSi in sediments from the Chinese marginal seas, it was also validated using sediments from the Chesapeake Bay, the Atlantic Ocean, and the Southern Ocean. The protocol can be used to quantify bSi in recently deposited and aged sediments from the Holocene period. The protocol contributes to the ongoing efforts to minimize the methodological bias that exist in bSi quantification and the bSi burial flux evaluation, thereby assisting in our understanding of Si cycling in the modern ocean.

• This protocol provides a step-by-step wet chemical extraction procedures and the measurement of dissolved Si in an alkaline solution using spectrophotometer.

• This protocol is easy to set up and reproduce, and determines bSi content with high precision.

• The protocol can be used to determine bSi in sediments of marginal seas and the open ocean.

## Graphical abstract

**Figure:**
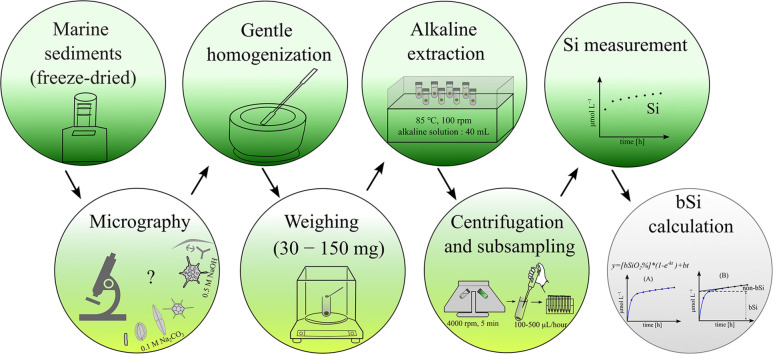

## Specifications table

**Table:** 

**Subject area**	Earth and Planetary Sciences
**More specific subject area**	Geochemistry, biogeochemistry, marine sciences
**Name of your protocol**	A wet chemical extraction protocol for measuring biogenic silica in marine sediments
**Reagents/tools**	While we provide the source of the materials used, the reagents and equipment listed in this box can be purchased from any supplier. **Reagents:** Sodium metasilicate (Na_2_SiO_3_·9H_2_O): Sigma-Aldrich S4392. Sodium hydroxide (NaOH): Sigma-Aldrich S5881. Sodium carbonate (Na_2_CO_3_): Sigma-Aldrich 223,530. Hydrochloric acid (HCl): Sigma-Aldrich 258,148. Hydrogen peroxide (H_2_O_2_): Nanjing Reagent C0404510123. Sulfuric acid (H_2_SO_4_): Sigma-Aldrich 258,105. Ammonium heptamolybdate tetrahydrate ((NH_4_)_6_Mo_7_O_24_·4H_2_O): Aladdin A597691. Oxalic acid (C_2_H_2_O_4_): Aladdin O107180. Metol (C_7_H_9_NO·0.5H_2_SO_4_): Aladdin M110716. Sodium sulfite (Na_2_SO_3_): Aladdin S112300. **Equipment:** Analytical balance (±0.1 mg): Cubis® II 224S-2CCN, Sartorius AG, Otto-Brenner-Straße 20, 37,079 Göttingen, Germany. Electric balance (±10 mg): YP502N, Shanghai Precision Instrument Co., Ltd, Shanghai, China. Water Purification System: Elix® Reference water purification system, Merck Millipore, EMD Millipore Corporation, Billerica, MA, U.S.A. Freeze drier: Alpha 1–2 LSCbasic, Martin Christ, Gefriertrocknungsanlagen, An der Unteren Söse 50, 37,520 Osterode am Harz, Germany. Centrifugal machine: Multifuge 3SR+, Thermofisher Scientific, Thermo Electron LED GmbH, D-37,520, Osterode am Harz, Deutschland. Water bath: Julabo SW22, JULABO GmbH, Gerhard-Juchheim-Strasse 1, 77,960 Seelbach, Germany. Mixer: DLAB MX-S, Dragon Lab, Beijing, China. Magnetic stirrer: B11–1, Shanghai Sile Instruments Co., Ltd., Shanghai, China. Pipette: Eppendorf Reference®2, Eppendorf SE Barkhausenweg 1, D-22,339, Hamburg, Germany. Ultraviolet-Visible Spectrophotometer: EU-2600A, Shanghai Onlab Instrument Co., Ltd., Shanghai, China. Microscope: Zeiss Axio Observer A1, Carl Zeiss AG, Carl-Zeiss-Straße 22, 73,447 Oberkochen, Germany. Desiccator: Shuniu, Sichuan Shubo group Co., Ltd. Auto-analyzer: AA3 HR auto-analyzer, SEAL Analytical. ICP-OES: iCAP6300, Thermo Scientific. Centrifuge tube: 50 mL fluorinated ethylene propylene (FEP) centrifuge tube, Nalgene no. 3114–0050, Caps no. DS3131–0024; 50 mL polypropylene copolymer centrifuge tube, Nalgene no. 3119–0050, Caps no. DS3132–0024; 15 mL polypropylene (PP) Conical Centrifuge Tube, Axygen®, Corning. Lab spatula: Merck HS15909.
**Experimental design**	The types of biogenic silica (bSi) present in freeze-dried sediments were identified under a microscope to assist in selecting appropriate alkaline solution. After gentle homogenization using an agate mortar and lab spatula, the sediments were weighed into a conical centrifuge tube and filled with alkaline solution. The tube was then soaked in a preheated water bath to speed up the dissolution of bSi. Subsampling of the alkaline solution was conducted at one-hour intervals after centrifugation. The alkaline-leachable silicon content was measured using a UV spectrophotometer, and the bSi content was evaluated based on the time-course dissolution of silica.
**Trial registration**	N/A
**Ethics**	N/A
**Value of the Protocol**	This protocol provides a detailed description of wet chemical extraction procedures and subsequent measurement of alkaline-leachable silicon concentrations. It is easy to reproduce and provides a standardized, high-precision method for determining biogenic silica content. It can be used to determine biogenic silica content in sediments ranging from marginal seas to the open ocean.

## Background

Biogenic silica (bSi), primarily derived from siliceous microfossils such as diatoms, radiolarians, and sponge spicules, constitutes a critical component of marine biogeochemical cycles and climate regulation [1,2]. As the primary repository of dissolved silicon (Si) in oceanic systems, bSi serves as a proxy for reconstructing past productivity, assessing modern carbon sequestration mechanisms, and understanding the intricate coupling of Si and carbon cycles in marginal seas and open ocean environments [3,4].

Accurate quantification of bSi in marine sediments is indispensable for elucidating historical changes in marine productivity, evaluating anthropogenic impacts on nutrient dynamics, and predicting future responses of marine ecosystems to climatic perturbations [2,5]. However, conventional wet chemical extraction methods—often relying on standardized alkaline solutions (e.g., 0.1–0.5 M Na₂CO₃/NaOH) and fixed digestion times—face significant challenges when applied to heterogeneous marine sediments. These include incomplete dissolution of resistant bSi phases (e.g., radiolarians and sponge spicules), co-dissolution of lithogenic silicates, and matrix interference from organic matter or authigenic minerals, leading to systematic biases in bSi burial flux estimates [5,6].

Existing protocols, such as the widely adopted 5-hour DeMaster (1981) method [7], lack flexibility to address sediment-specific complexities. For instance, sediments rich in radiolarians or sponge spicules require prolonged extraction (>8 h) and stronger alkalis (e.g., 0.5 M NaOH) to maximize bSi recovery, while diatom-dominated samples risk overestimation due to aluminosilicate dissolution under aggressive conditions [[5], [6], [7]]. Additionally, traditional homogenization techniques (e.g., powdering) accelerate lithogenic silicate dissolution, further compromising accuracy [5]. These limitations are exacerbated in marginal seas (e.g., river-influenced East China Sea) and open-ocean settings (e.g., Southern Ocean siliceous oozes), where sediment composition varies widely.

To address these gaps, we present an optimized wet chemical extraction protocol that integrates sediment-specific alkaline solution selection, kinetic dissolution modeling, and rigorous quality control. Key innovations include: microscopy-guided alkaline solution selection, extended extraction kinetics, standardized dissolved Si quantification, and dual bSi calculation methods. Validated across diverse Holocene sediments—from eutrophic estuaries (Chesapeake Bay) to oligotrophic open oceans—this protocol achieves 70 % higher bSi recovery in radiolarian-rich sediments compared to conventional Na₂CO₃ extractions. Its reproducibility (triplicate RSD <5 %) and adaptability to low-bSi open-ocean sediments (<0.5 % SiO₂) address critical gaps in global bSi burial flux assessments. By harmonizing methodological rigor with practical accessibility, this protocol advances the fidelity of marine Si cycle studies and supports emerging research in climate-biogeochemistry interactions.

## Description of protocol

### Sample preparation

Sample preparation includes drying the sediments, micrography, sediment homogenization, and weighing.

1. Freeze drying

Approximately 200 g of wet sediments are stored at −20 °C for 12 h, and then dried in a freeze dryer for 48–72 h until dry. Freeze-drying is recommended, as oven drying can potentially cause fragmentation of the biosiliceous structure [8].

2. Micrography

Approximately 5 mg of dried sediment was reacted with 1 mL of 1.0 M HCl and 1 mL of 30 % H_2_O_2_ for two h in a 15 mL centrifuge tube. The aim was to remove the organic matter and calcareous skeletons, leaving the bSi and lithogenic silicate in the residual. The residual was then washed three times with 5 mL of Milli-Q water, then mixed with an additional 1 mL of Milli-Q water using an electrical mixer (1000 rpm, 10 s). The well-mixed samples were transferred onto a glass microscope slide using a pipette. Different types of bSi, i.e., phytolith, diatoms, sponge spicules, radiolarians, and silicoflagellates, were identified using an optical microscope.

**Fig. 1:**
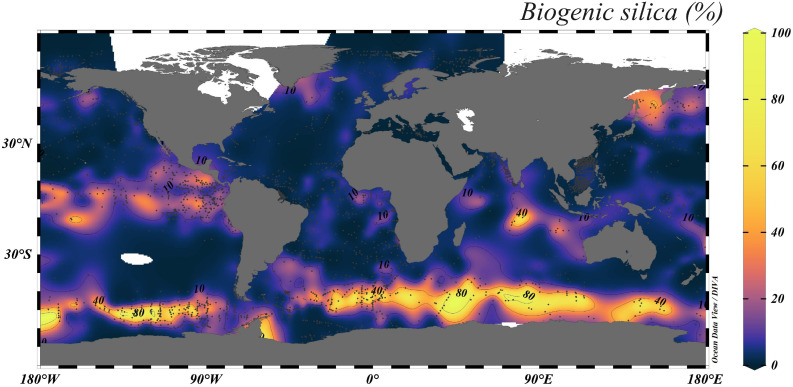
Biogenic silica content (SiO_2_ %) in surface sediments of the global ocean. This figure was created using previously published data [5,9,10]. The white area represents data that are not available. The figure was plotted using Ocean Data View (ODV) program [11].

Samples containing mainly diatoms, phytoliths and silicoflagellates (90 % of the total bSi abundance) require wet chemical extraction (8 h) using 0.1 M Na_2_CO_3_ solution. Samples containing an important abundance of sponge spicules and radiolarians (>10 % of the bSi abundance), or with a bSi content larger than 10 %, require digestion (10−12 h) using a 0.5 M NaOH solution. The bSi content of the sediment can be estimated approximately in accordance with Fig. 1 in order to select the appropriate alkaline solution.

3. Homogenization of sediments and weighing

The freeze-dried sediments were gently homogenized using an agate mortar and lab spatula. This procedure aims to improve the reproducibility of bSi measurements by mixing the sediments, while minimize the destruction of the physical structures of biosiliceous skeletons and lithogenic silicates. Powdering the sediment can cause the destruction of bSi and lithogenic silicates, thus enhancing their dissolution when exposed to alkaline solution [12]. To reduce the interference of lithogenic silica dissolution on bSi quantification, it is not recommended to powder sediments containing high levels of lithogenic silicates. The well-homogenized sediments were stored in a desiccator at room temperature.

The sediments were weighed using an analytical balance. A 50 mL fluorinated ethylene propylene (FEP) or polypropylene copolymer centrifuge tube was placed on the balance, and 30–150 mg of homogenized freeze-dried sediment was added to the centrifuge tube. Weighing the samples in a centrifuge tube can minimize the loss of sediment material. The mass of sediment (e.g., 30−150 mg) is designed for an effective extraction of bSi and for the analysis of dissolved Si concentration (see Table 1). Here, the maxim concentration of Si in alkaline solution is approximately 1000 μmol L^−1^, which is much lower than the Si concentration (9000 μmol L^−1^) proposed by Mortlock and Froelich [13].

**Table 1:** An example showing sediment mass, biogenic silica content, and the Si concentration in an alkaline solution and a working solution. The Si concentration in the alkaline solution was designed to be lower than 1000 μmol L^−1^, and the Si concentration in the working solution (after dilution by a factor of 75) was lower than 15 μmol L^−1^. Therefore, a working standard solution ranging from 0 to 20 μmol L^−1^ is suitable for quantifying the dissolved Si concentration in leachate.

Mass of sediments [mg]	Biogenic silica [ %SiO_2_]	Si in sediment [μmol]	Volume of alkaline solution [mL]	Si concentration in alkaline solution [μmol L^−1^]	Estimated Si concentration in working solution [μmol L^−1^]
30	5.0	2.5	40	62.5	0.83
30	10.0	5.0	40	125.0	1.67
30	20.0	10.0	40	250.0	3.33
30	30.0	15.0	40	375.0	5.00
30	50.0	25.0	40	625.0	8.33
30	80.0	40.0	40	1000.0	13.33
150	0.1	0.3	40	6.3	0.08
150	0.5	1.3	40	31.3	0.42
150	1.0	2.5	40	62.5	0.83
150	3.0	7.5	40	187.5	2.50
150	5.0	12.5	40	312.5	4.17
150	10.0	25.0	40	625.0	8.33

### Wet chemical extraction

**Fig. 2:**
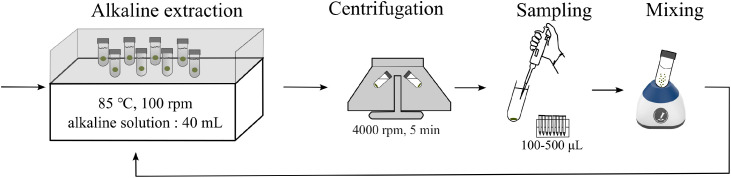
Schematic diagram shows the alkaline extraction procedure.

Fig. 2 shows the wet chemical extraction process. Here, 40 mL of an alkaline solution (0.1 M Na_2_CO_3_ or 0.5 M NaOH, depending on the bSi content and types) was added to a centrifuge tube containing pre-weighed sediments. The solution and sediments were then mixed using an electric mixer (1000 rpm, 30 s). After sealing the tubes tightly with caps, they were placed in a water bath that had been pre-heated to 85 °C and shaken at a frequency of 100 rpm. At each one-hour time interval, the tube was immediately centrifuged at 4000 rpm for 5 min, after which 200 μL of leachate was taken using a pipette for dissolved Si analysis. Depending on the concentration of dissolved Si in the leachate, this volume can be adjusted between 100 and 500 μL. The leachate was stored in a 15 mL polypropylene conical centrifuge tube at 4 °C before measurement. In addition, the sediment and alkaline solution were mixed well using an electric mixer (1000 rpm, 30 s) after subsampling, after which the tubes were placed in the hot shaking water bath again. The period of extraction is suggested to be 8 h [14], which is three h longer than the traditional 5-hour exaction proposed by DeMaster [7]. Moreover, it is recommended that sediments containing important amounts of sponge spicules and radiolarian skeletons be extracted for 10–12 h.

### Measurement of dissolved silicon concentration

The dissolved Si concentration was determined using a molybdenum blue photometric method with an Ultraviolet-Visible (UV) spectrophotometer [5]. Prior to measuring the dissolved Si, the reagents listed in Table 2 were prepared. The alkaline leachate (0.5 M NaOH, 200 μL) was neutralized using an HCl solution (100 μL of 1.0 M HCl), and the Si working standards were prepared according to Table 3. The solution matrix of the dissolved Si working standard is similar to that of the alkaline leachate solution. The neutralized solution was then diluted with Milli-Q water to 10 mL. Then, 2 mL of acidified ammonium molybdate solution was added to the sample and mixed. After 20 min, 3 mL of chromogenic reagent (reagent E in Table 2) was added to the tube. The mixture solution (15 mL) was thoroughly mixed using an electric mixer (1000 rpm, 10 s). After three h, the standards and samples were analyzed using the UV spectrophotometer (λ = 810 nm, 1 cm cuvette). Note that the UV spectrophotometer should be switched on 30 min prior to measurement, and that the standards should be analyzed before the sediment leachate samples. A coefficient of determination (R^2^) for the standard curve greater than 0.9995 is preferred. Furthermore, the concentration of dissolved Si in the reagent blank solutions needs to be evaluated [10].

**Table 2:** Preparation of reagents for dissolved silicon colorimetric reaction.

Reagents	Operating instructions	Storage condition
A: Saturated oxalic acid (10 % C_2_H_2_O_4_)	Dissolve 100 g of C_2_H_2_O_4_ in 1 L of Milli-Q water.	Store at 4 °C in a refrigerator, and it will remain stable for one year.
B: Sulfuric acid solution (H_2_SO_4_: H_2_O = 1:2)	Mix 250 mL of concentrated H_2_SO_4_ with 500 mL of Milli-Q water.	Store at room temperature, and it will remain stable for one year.
C: Acidified ammonium molybdate	Dissolve 8.0 g of ammonium molybdate in 976 mL of Milli-Q water in a brown plastic bottle. Then, add 24 mL of concentrated HCl solution.	Store at 4 °C in a refrigerator under dark conditions, and it will remain stable for one month.
D: Metol-Sodium sulfite solution	Dissolve 20 g of C_7_H_9_NO · 0.5H_2_SO_4_ in 800 mL of Milli-Q water, then add 12 g of Na_2_SO_3_. Once the sodium sulfite has fully dissolved, add a further 200 mL of Milli-Q water.	Store at 4 °C in a refrigerator under dark conditions, and it will remain stable for one month.
E: Chromogenic reagent	60 mL A solution + 90 mL B solution + 100 mL D solution + 50 mL Milli-Q water	Prepare the solution before use.
Silicon stock solution (20.0 mmol L^−1^)	Dissolve 5.684 g of Na_2_SiO_3_·9H_2_O in 500 mL of Milli-Q water in a 1 L plastic volumetric flask. Once the Na_2_SiO_3_·9H_2_O has fully dissolved, top up with Milli-Q water to reach the 1 L mark.	Store at 4 °C in a refrigerator under dark conditions, and it will remain stable for six months.
Silicon standard solution (100 μmol L^−1^)	Transfer 0.5 mL of the silicon stock solution to a 100 mL plastic volumetric flask, and top up with Milli-Q water until the 100 mL mark is reached.	Prepare the standard solution before use.

**Table 3:** An example of a dissolved Si working standard. In this study, a solution matrix similar to that of the standards and sediment leachate (0.5 M NaOH) was created, with a final sample mixture solution volume of 10 mL prior to the chromogenic reaction. This volume can be adjusted to 10 mL, 25 mL or 50 mL according to the volume of colorimetric tube and the concentration of dissolved silicon in a specific study. Those who are unfamiliar with chemical analysis are advised to analyses the standard tens of times.

Number	Concentration of Si [μmol L^−1^]	Volume of Si standard solution [μL]	Volume of alkaline solution (0.5 M NaOH) [μL]	Volume of acid (1.0 M HCl) used for neutral reaction [μL]	Volume after addition of Milli-Q water [mL]	Volume of acidified ammonium molybdate [mL]	Volume of Chromogenic reagent [mL]
1	0	0	200	100	10	2	3
2	0.25	37.5	200	100	10	2	3
3	0.50	75.0	200	100	10	2	3
4	0.75	112.5	200	100	10	2	3
5	1.00	150.0	200	100	10	2	3
6	3.00	450.0	200	100	10	2	3
7	5.00	750.0	200	100	10	2	3
8	10.00	1500.0	200	100	10	2	3
9	15.00	2250.0	200	100	10	2	3

The Si concentration of leachate was calculated based on the linear relationship between the Si concentration (unit: μmol L^−1^) and absorbance value (unit: 1) of Si working standards. We compared the dissolved Si measurements obtained using the UV spectrophotometer, a nutrient auto-analyzer, and an ICP-OES. The results obtained using these methods are comparable (see Fig. 5 in the Protocol Validation section). This protocol was chosen because it uses a UV spectrophotometer to determine the dissolved Si concentration, which is cost-effective and can measure up to 400 liquid samples (approximately 50 sediment samples) in one day. Note that the Si concentrations in leachate can be converted into SiO_2_ % (see Eq. 1). As shown in Eq. 1, *C, V* and *M* represent the Si concentration, the volume of leachate and the mass of the sediment, respectively.

(Eq. 1) $$SiO_{2}\%=100\;\times \left[10^{-6}\times C\;\left(\mu mol\;L^{-1}\right)\times V\;\left(L\right)\times \;60\;\left(g\;mol^{-1}\right)/\left(M\left(g\right)\right)\right]\;$$

### Calculation of biogenic silica content

**Fig. 3:**
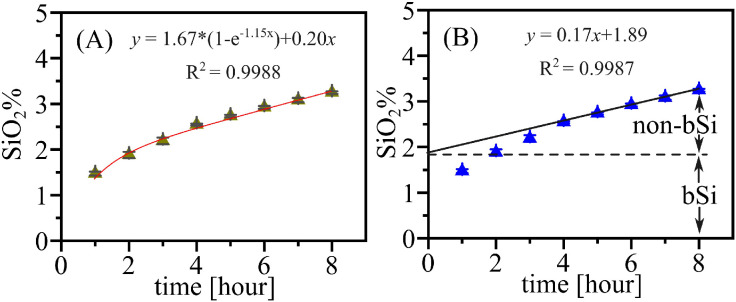
Examples of bSi content (SiO_2_ %) calculated using two methods. A, non-linear fitting using a first-order dissolution kinetic model (see Eq. 2); B, linear regression of the linearly dissolving phases (e.g., the last four data points). The figures were plotted based on the alkaline extraction of sample C12 from the East China Sea, performed in triplicate. The bSi content was found to be 1.71 ± 0.04 %, and 1.89 ± 0.02 % using methods A and B, respectively.

This section describes two commonly used methods for calculating the biogenic silica content: A) calculating the bSi content using a first-order dissolution kinetic model (see Eq. 2, and Fig. 3A) [15,16]; and B) a linear regression calculation based on the Si vs. time curve (see Fig. 2B) [7,14,17].

**Fig. 4:**
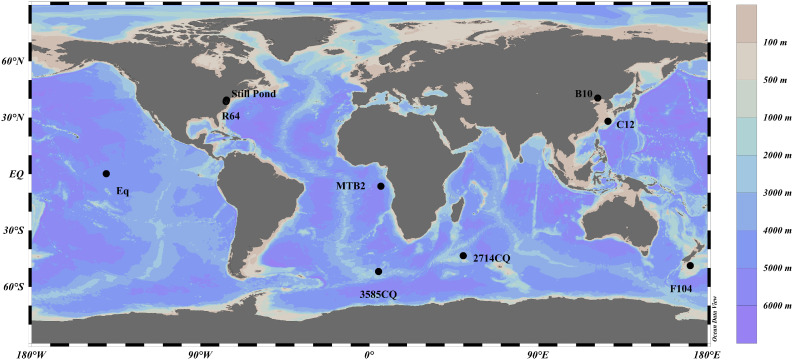
Location of eight sediment samples used for protocol validation. The samples are surface sediment (0 – 2 cm) obtained from marginal seas (e.g., B10, C12, F104, Still Pond, and R64) and the open ocean (MTB2, 3585CQ, and 2714CQ). The water depth at the sites where these samples were collected ranges from 10 m to 5000 m.

**Method A:** In Eq. 2, [bSiO_2_ %] represents the bSi content, *y* and *t* are the concentrations of Si (SiO_2_ %) in leachate and the extraction time (hours), *k* and *b* represent the apparent rate constants in the alkaline solution that summarize the impact of biogenic and lithogenic silica on the dissolved Si concentration respectively [16]. This calculation was conducted using Origin 2021b software in the current study, but it can also be performed using other programs (i.e., Solver in Excel, MATLAB or Python).
  (Eq. 2) $$y=\left[bSiO_{2}\%\right]*\left(1-e^{-kt}\right)+bt$$
**Method B:** A linear regression was calculated based on the last 3–4 data points due to their linearly dissolving characteristics (see Fig. 4B), and the intercept was considered as bSi content. This method is the most commonly applied in previous studies [5,7,18]. However, it should be stated that this method is not recommended, as the selection of data points for linear extrapolation is highly subjective.

### Protocol validation

**1. Materials**

The protocol was validated using eight sediment samples that were obtained from marginal seas and the open ocean. Additionally, this study determined two samples from a previous international intercomparison campaign [19]: sample Still Pond and R64 (see Fig. 4). Sample F104 was obtained off the coast of New Zealand. Samples B10 and C12 were obtained from the Bohai Sea and the East China Sea, respectively [5]. Samples Still Pond and R64 were obtained from the Chesapeake Bay [19]. Sample MTB2 was obtained from the Congo deep-sea fan (>4000 m water depth) during the Western Atlantic Climate Study (WACS) cruise [20]. Samples 2714CQ and 3585CQ were obtained from the siliceous ooze in the Southern Ocean. Following the protocol, all the samples were freeze-dried and gently homogenized using an agate mortar and lab spatula. The samples were then stored in a desiccator at room temperature before analysis.

**2. Microscopy and selection of alkaline solution**

Following microscopic observation, the predominant types of biosiliceous structure were identified (see Table 4). Sample B10 contains mainly diatoms, while samples F104 and C12 contain a mixture of diatoms, radiolarians and sponge spicules. Samples Still Pond, R64 and MTB2 mainly comprise diatoms and sponge spicules, and samples 2714CQ and 3585CQ mainly contain diatoms and radiolarians. According to the protocol, the alkaline solution recommended for each sample is listed in Table 4. Notably, this study conducted alkaline extractions using both 0.1 M Na_2_CO_3_ and 0.5 M NaOH in order to demonstrate the difference in bSi yield between the two extraction methods.

**3. Dissolved Si measurement**

**Table 4:** Types of biogenic silica in sediment samples and the proposed alkaline solution. “Y” and “N” represent the observation or non-observation of biosiliceous structures, respectively.

Sample code	Diatoms	Radiolarians	Sponge spicules	Alkaline solution
F104	Y	Y	Y	0.5 M NaOH
B10	Y	N	N	0.1 M Na_2_CO_3_
C12	Y	Y	Y	0.5 M NaOH
Still Pond	Y	N	Y	0.5 M NaOH
R64	Y	N	Y	0.5 M NaOH
MTB2	Y	N	Y	0.5 M NaOH
2714CQ	Y	Y	N	0.5 M NaOH
3585CQ	Y	Y	N	0.5 M NaOH

**Fig. 5:**
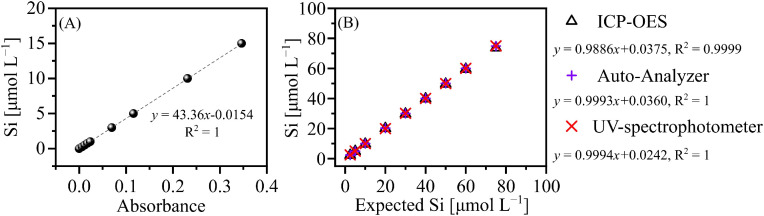
Si working standard curve (A) and comparison of dissolved Si concentrations determined using different methods (B). The absorbance of the Si working standards and corresponding dissolved Si concentration were determined using an UV-spectrophotometer (A). The Si concentrations in plot B were determined using an ICP-OES, a nutrient auto-analyser and a UV spectrophotometer.

Dissolved Si was measured using a molybdenum blue photometric method with a UV-spectrophotometer [5]. A Si working standard (Fig. 5A) was determined in accordance with the procedures outlined in Table 2 and Table 3. A linear relationship (R^2^ = 1) was found between the measured absorbance and Si concentration, demonstrating the reliability of the measurement. Additionally, measurements of dissolved Si obtained using an ICP-OES and a nutrient auto-analyser were compared with those obtained using a UV spectrophotometer (Fig. 5B). The results suggest that there is no obvious difference in the Si concentrations examined among the three techniques (Si concentration ranges from 0 to 80 μL^−1^). Therefore, reliable data can be obtained by measuring the dissolved Si of the alkaline leachate using the molybdenum blue photometric method with a UV spectrophotometer.

**4. Alkaline extraction and biogenic silica content calculation**

**Fig. 6:**
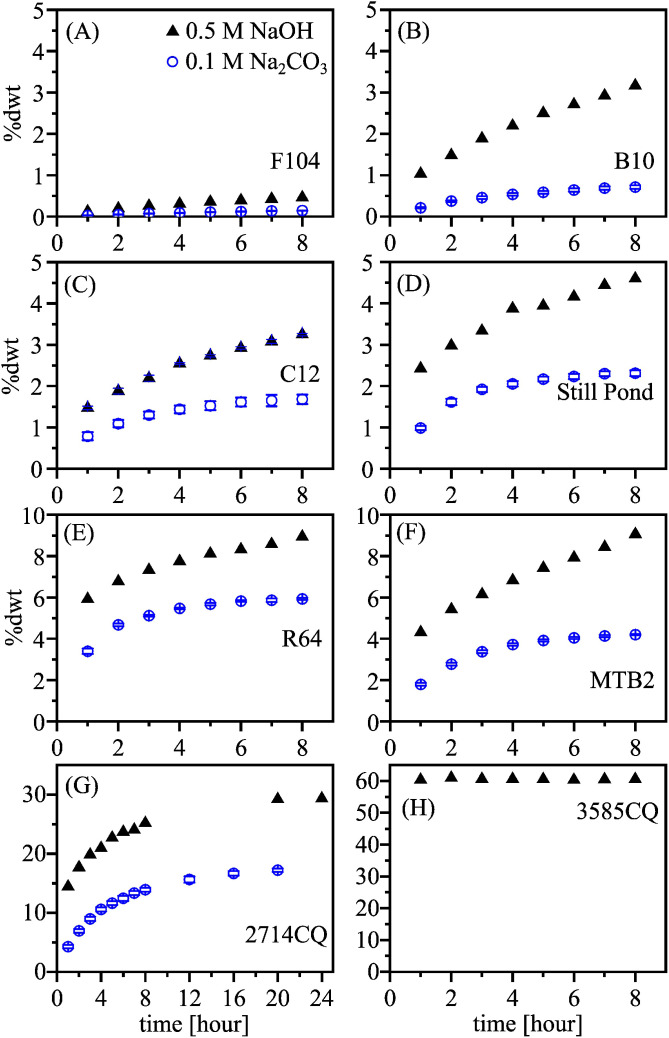
Silica (SiO_2_ %, dry weight/dwt %) extracted using a 0.1 M Na_2_CO_3_, and a 0.5 M NaOH solution. The error bars represent triplicate alkaline extractions. A) sample F104 from offshore New Zealand, B) sample B10 from the Bohai Sea, C) sample C12 from the East China Sea, D and E) sample Still Pond form the Chesapeake Bay, F) sample MTB2 from the Congo deep-sea fan, G and H) sample 2714CQ and 3585CQ from the Southern Ocean. The types of bSi in each sample were shown in Table 4. Note that the scales on the two axes are different.

Fig. 6 shows the sediment extraction using both 0.1 M Na_2_CO_3_ and 0.5 M NaOH. Triplicate alkaline extractions were performed in order to test the reproducibility of the protocol. The silica content extracted using 0.5 M NaOH was higher than that extracted using 0.1 M Na_2_CO_3_ (Fig. 6), indicating that 0.5 M NaOH is capable of extracting more resistant biogenic silica and lithogenic silicate than the 0.1 M Na_2_CO_3_ solution. Of all the samples, the silica content extracted using alkaline solution was lowest in sample F104 (approximately 0.5 %, see Fig. 6A), and the highest in sample 3585CQ (approximately 60 %, see Fig. 6H). The alkaline-extractable silica content of sample B10, C12 and Still Pond was lower than 5 % (Fig. 6B−D), and lower than 10 % for samples R64 and MTB2 (Fig. 6E−F). Notably, the silica extracted using 0.5 M NaOH was 70 % higher than that extracted using 0.1 M Na_2_CO_3_ (29.37 % vs. 17.21 %), highlighting the importance of selecting the appropriate alkaline solution based on micrography.

The bSi contents were analysed using the two methods described in this protocol (Table 5). Based on a two-way analysis of variance (ANOVA), no significant difference was observed between the two methods (0.1 M Na_2_CO_3_ extraction: *p* = 0.154, 0.5 M NaOH extraction: *p* = 0.585). However, the bSi yield from the 0.5 M NaOH extraction was significantly higher than that from the 0.1 M Na_2_CO_3_ extraction (*p* = 0.01; Table 5). The “**estimated bSi contents**” shown in Table 5 were calculated using the recommended alkaline solution for the eight samples (see Table 4 for sample information). This value is considered as an appropriate sediment bSi content due to the rigorous analytical procedure and the quality control measures during sample preparation, alkaline extraction, dissolved Si measurement and the mathematical calculation.

**Table 5:** Evaluation of the biogenic silica content (SiO_2_ %). The errors in values marked with “a” were calculated using modelling, while errors in other values were calculated based on triplicate extractions. N/A indicates that data are unavailable because the sample did not undergo Na_2_CO_3_ extraction.

Sample	0.1 M Na_2_CO_3_ extraction		0.5 M NaOH extraction		Estimated bSi content
	Calculation method A	Calculation method B	Calculation method A	Calculation method B	
F104	0.19 ± 0.07	0.04 ± 0.01	0.19 ± 0.02^a^	0.18	0.19 ± 0.02
B10	0.48 ± 0.05	0.38 ± 0.02	1.31 ± 0.10^a^	1.39	0.48 ± 0.05
C12	1.28 ± 0.01	1.29 ± 0.08	1.71 ± 0.04	1.89 ± 0.02	1.71 ± 0.04
Still Pond	2.19 ± 0.02	1.93 ± 0.05	2.78 ± 0.17^a^	2.82	2.78 ± 0.17
R64	5.19 ± 0.14	5.30 ± 0.09	6.43 ± 0.13^a^	6.73	6.43 ± 0.13
MTB2	3.99 ± 0.07	3.47 ± 0.11	4.52 ± 0.01^a^	4.70	4.52 ± 0.01
2714CQ	13.60 ± 0.47	12.02 ± 0.35	22.59 ± 0.65^a^	22.35	22.59 ± 0.65
3585CQ	N/A	N/A	60.62 ± 0.79^a^	60.40	60.62 ± 0.79

**Fig. 7:**
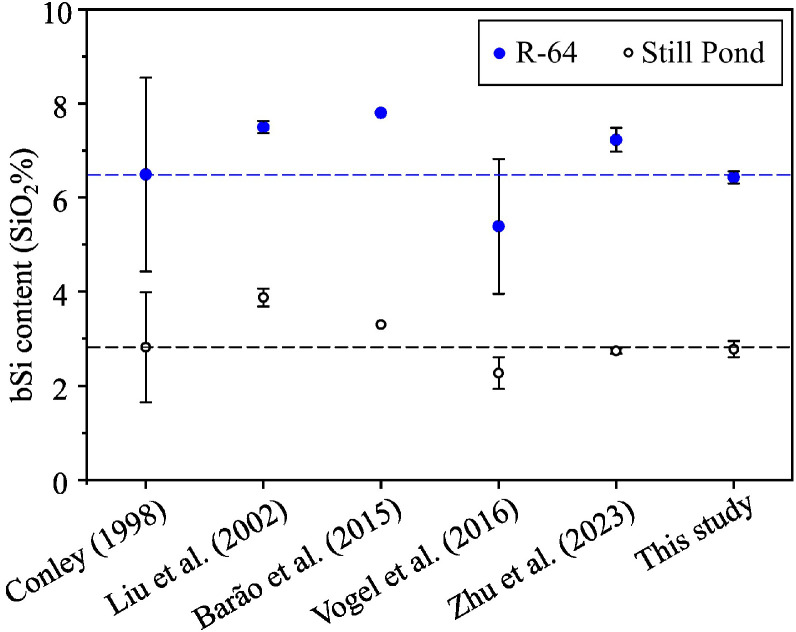
Comparation of the biogenic silica content (SiO_2_ %) of sample Still Pond and R-64 determined in this study and the previously published data [5,13,19,22,23]. The blue and grey dished lines represent the mean bSi value of sample R-64 and Still Pond, respectively.

Furthermore, this study determined the bSi content of Still Pond and R-64 (samples from the inter-laboratory comparison campaign) in accordance with our proposed protocol. These bSi values were then compared with the data presented in Conley (1998) [19] and other study [5] (see Fig. 7). Our results (Table 5) are consistent with the inter-laboratory comparison data (Still Pond: 2.82 ± 1.17 %, R-64: 6.49 ± 2.06 %) [19], but are smaller than the bSi content determined by extracting powdered sediments with a 2.0 M Na_2_CO_3_ solution [13] or a measurement that did not display replicate extractions [22]. As presented in Conley (1998) [19], a significant discrepancy in the bSi values occurs when different alkaline extraction techniques are employed (i.e., sample preparation, alkaline solution and concentration, and mineral correction): 41 % for Still Pond and 32 % for R-64. Powdering of sediment destructs the structure of silicate minerals and enhances the dissolution of lithogenic silicates, thus overestimating the bSi content. Therefore, this protocol utilizes an agate mortar and lab spatula to homogenize freeze-dried sediments instead of grinding them into powder. Fig. 7 also shows that the bSi values determined in this study are higher than those obtained by alkaline extraction for 5 h [23]. This may be due to the incomplete extraction of resistant bSi (e.g., sponge spicules) presented in the samples [5]. This emphasizes the necessity of knowing the types of bSi present in sediments prior to selecting an appropriate alkaline extraction method (e.g., alkaline solution, and extraction time).

### Limitations

The samples used in this study are Holocene sediments, which are less than 11,700 years old. Biosiliceous structures in sediments that are older than the Holocene (i.e., the Miocene or Paleocene, which are 2−24 million years old) may undergo alteration and transformation (e.g., transformation from amorphous silica/opal-A to crystalized silica/opal-CT). These structures are therefore more difficult to fully extract using the alkaline concentration presented in this protocol. Future research involving Miocene − Paleocene (or even older) sediments should use strong alkalis (e.g., 2.0 M NaOH or KOH) to leach these physically altered biosiliceous structures for 9−12 h [21], in accordance with our protocol. Moreover, the diagenesis and alternation of bSi will be enhanced under a high geothermal gradient [24]. Therefore, measuring bSi in sediments with a high geothermal gradient may require strong alkaline extraction or an extended extraction time.

## Related research article

D. Zhu, J.N. Sutton, A. Leynaert, P.J. Tréguer, J. Schoelynck, M. Gallinari, Y. Ma, S.M. Liu, Revisiting the biogenic silica burial flux determinations: A case study for the East China seas. Front. Mar. Sci. 9 (2023) 1058,730. https://doi.org/10.3389/fmars.2022.1058730

## Supplementary material *and/or* additional information [OPTIONAL]

Not applicable.

## CRediT authorship contribution statement

**Dongdong Zhu:** Conceptualization, Methodology, Software, Validation, Formal analysis, Investigation, Data curation, Writing – original draft, Visualization, Funding acquisition. **Su Mei Liu:** Conceptualization, Methodology, Investigation, Resources, Writing – review & editing, Supervision, Project administration, Funding acquisition. **Aude Leynaert:** Conceptualization, Methodology, Investigation, Resources, Writing – review & editing, Supervision, Project administration, Funding acquisition. **Paul Tréguer:** Conceptualization, Methodology, Investigation, Resources, Writing – review & editing, Supervision, Project administration, Funding acquisition. **Morgane Gallinari:** Conceptualization, Methodology, Investigation, Resources, Writing – review & editing, Supervision, Project administration, Funding acquisition. **Heting Zhou:** Investigation, Resources, Writing – review & editing. **Jill N. Sutton:** Conceptualization, Methodology, Investigation, Resources, Writing – review & editing, Supervision, Project administration, Funding acquisition.

## Declaration of competing interest

The authors declare that they have no known competing financial interests or personal relationships that could have appeared to influence the work reported in this paper.

## Data Availability

Data will be made available on request.
